# Reflections and perspectives on epigenetically mediated biological control: compromises in cancer and skeletal pathology

**DOI:** 10.20935/acadbiol7628

**Published:** 2025-04-11

**Authors:** Gary S. Stein

**Affiliations:** 1 Department of Biochemistry, Larner College of Medicine, University of Vermont, 89 Beaumont Avenue, Burlington, VT 05405, USA.; 2 University of Vermont Cancer Center, Larner College of Medicine, University of Vermont, 89 Beaumont Avenue, Burlington, VT 05405, USA.

**Keywords:** epigenetic control, cell cycle, mitotic bookmarking, bhromatin, nuclear organization, transcription, histone code, stem cells, cancer hallmarks, bone biology and pathology

## Abstract

Establishing DNA as the genetic material, deciphering the genetic code and defining the double helical structure of the genome provided a foundation for pursuing a mechanistic understanding of gene expression that is operative for biological control and pathology. Equally important are a series of discoveries that have established epigenetic mechanisms, defined as the control of gene expression by non-DNA-encoded regulation, mediated by an evolving histone code that supports chromatin states, as complementary and obligatory determinants for biological activity. Evidence is accruing for pivotal contributions by epigenetic parameters of control to gene expression that is required for development and differentiation as well as for transient and sustained responsiveness to physiological regulatory cues that support cell structure, function, cell survival, tissue repair and remodeling. Epigenetic control is emerging as a decisive contributor to the regulatory compliance for the highly integrative processes that balance phenotypic function and tumor suppression with aberrant gene expression that accompanies the onset and progression of disease, strikingly illustrated with tumorigenesis and skeletal disorders.

## 1. Introduction

This paper presents an overview of the evolving understanding for the components and multiple dimensions that are operative in epigenetic regulation that is supported by the evolving engagement of the histone code that supports chromatin states controlling biological regulation and pathology [1–12]. The integration of genetic and epigenetic mechanisms will be reviewed. Emphasis will be on gene expression within the three-dimensional context of nuclear architecture, incorporating a fourth dimension, consideration for the dynamic contribution of synergy between space and time. The regulatory priority for the control of gene expression in nuclear microenvironments will be addressed with consideration for intranuclear trafficking that has been implicated in the organization, localization and assembly of regulatory complexes in non-membrane-bound nuclear compartments [13, 14], with the potential for the involvement of liquid phase separation as a regulatory component [15, 16]. Given the requirement for the persistence of epigenetic control in proliferating cells, the role of mitotic gene bookmarking to sustain epigenetic regulatory machinery that is operative in parental cells for the control of gene expression in progeny cells following cell division will be discussed (reviewed in [17–23]).

## 2. Engagement in defining epigenetic regulatory mechanisms

As investigators who initially cloned the human histone genes [24– 26], our laboratory had the opportunity to contribute to the development of concepts and experimental approaches for the epigenetic control of gene expression. We will share perspectives, challenges and advances that have shaped our current perspectives of epigenetic control with the caveat that the field is rapidly evolving. Understanding epigenetically modified biological control is accelerating, and targeting cancer-compromised epigenetic regulation is increasingly effective [27–31].

Trained in biology, physiology and pathology, I have a longstanding dedication to translating mechanistic understanding of cancer and skeletal biology to clinical applications. Transdisciplinary team research has always been the guiding strategy and the operational fabric for our research group. The research team has combined molecular, cellular, genomic and epigenomic experimental approaches with multispectral imaging, proteomic and spatial transcriptomic approaches. Emphasis has been placed on addressing the regulatory complexities of biological control with a priority to accelerate the translation of disease-compromised mechanisms to advances in the prevention, early detection, treatment and survivorship of cancer and skeletal disease. One of the central themes of our scientific research has been to establish mechanisms controlling proliferation and differentiation, with a focus on genetic and epigenetic regulatory networks that are obligatory for physiologically responsive control and are aberrant with the onset and progression of cancer. A long-standing collaboration with Janet Stein has resulted in pioneering contributions to mechanisms, molecular signatures and biomarkers, including those driven by intra- and inter-chromosomal interactions [32–35] and noncoding RNAs [36–43]. These initiatives have been directed toward advancing capabilities for the prevention, early detection, treatment and survivorship of breast and prostate cancer and leukemia as well as the mechanistic and clinical understanding of solid tumor metastasis to bone. Our laboratory has consistently been at the forefront of characterizing genetic and epigenetic regulation that mediates cell cycle control in lineage-committed cells, cancer stem cells and pluripotent and induced pluripotent stem cells with abbreviated cell cycle. The Stein lab has made pivotal contributions to understanding the transcriptome by defining mechanisms that govern the combinatorial organization, integration and assembly of regulatory machinery in nuclear microenvironments, higher-order genomic organization and the epigenetic control of cell fate and lineage commitment in biological control and cancer. The discovery of mitotic gene bookmarking [17–20, 44–47] and mitosis-specific bivalent histone modifications in pluripotent stem cells, which are recapitulated in early-stage breast and prostate cancer cells, establishes oncofetal epigenetic control as a novel dimension to tumorigenesis [48–50].

A decisively significant and consistently rewarding partnership with Janet Stein and longstanding sustained collaborations with Andre Van Wijnen, Jane Lian, Jonathan Gordon, Prachi Ghule, Sayeed Zaidi, Martin Montecino and Andrew Fritz have been exceptionally productive and personally gratifying. Mentors and colleagues have provided immense perspective and have been instrumental in sharing guidance in the development and implementation of concepts, strategies and experimental approaches. Beginning in the 1960s, Sheldon Penman was unquestionably the driving force and motivation for developing molecular and cellular approaches to experimentally address the components of cell structure–gene expression relationships. These studies established a foundation for mechanistically characterizing gene expression within the three-dimensional context of nuclear architecture. It was the recognition of the dynamic plasticity of gene organization that reinforced the importance of non-DNA-encoded regulatory information and the obligatory role of epigenetic control for the fidelity of cell structure and function as well as physiological responsiveness in transient, short-term and sustained cellular requirements.

Arthur Pardee, Renato Besarga and my PhD thesis advisor Howard Rothstein provided our research group with guidance and perspective to experimentally pursue regulatory mechanisms that govern cell cycle control, with an emphasis on the integration of regulatory machinery that is assembled in nuclear microenvironments for the support of combinatorial control and responsiveness to multi-dimensional signaling pathways and regulatory networks. Collaboration with Carlo Croce has been the basis for a cancer genetic dimension to our work as well as for the pursuit of noncoding RNAs, microRNAs, tsRNAs and long noncoding RNAs as epigenetic mechanisms of tumor suppression and tumor promotion. Sidney Winehouse was a valuable resource for insight into relationships between biochemical and molecular mechanisms that are obligatory in tumor biology and pathology. Dario Altieri was a driving force for exploring cancer-compromised metabolic control within the context of epithelial-to-mesenchymal transition and tumor progression.

Extensive collaboration with Scott Hiebert has leveraged the combined power of molecular and imaging approaches to investigate gene expression within the context of nuclear organization. These studies established a novel dimension to epigenetic control that includes the intra-nuclear trafficking of transcription factors and mitotic gene bookmarking and the retention of phenotype-determining regulatory factors at target gene loci on mitotic chromosomes to sustain competency for gene expression in progeny cells following cell division. Ongoing collaborations with Scott Hiebert and Kristy Stangel are utilizing a degron approach to investigate the requirement of mitotic bookmarking to epigenetically control tumor suppressor and tumor promoter activities. Degron technology involves the CRISPR introduction of a short amino acid sequence into a regulatory protein that, when activated by a small molecule, rapidly and selectively supports ubiquitin-mediated proteolytic degradation [51, 52].

Expanding the pursuit of epigenetic regulation, we have been addressing the contributions of higher-order chromatin organization. With Tony Imbalzano, we examined the involvement of the chromatin remodeling machinery in modulating gene expression that is required for proliferation and phenotype commitment with epigenetic consequences following transformation and tumorigenesis. Appreciating the epigenetic power of higher-order chromatin organization to integrate the activities of contiguous and distal genomic elements as well as establish regulatory boundaries, we have been collaborating with Job Dekker and Tom Misteli to identify and functionally characterize the pivotal role of genomic organization in epigenetically driven biological control and pathology.

Establishing the epigenetic landscape for the histone gene loci provided direct evidence for the regulatory dynamics of higher-order chromatin organization that is focally confined to nuclear microenvironments designated as histone locus bodies for the support of cell cycle stage-specific gene expression at the G1/S phase transition. This high-resolution strategy permits the comprehensive, genome-wide mapping of noncontiguous chromatin interactions. The approach involves formaldehyde cross-linking, followed by the digestion and relegation of covalently linked DNA fragments, providing a direct readout of the genomic landscape, reflecting the physical three-dimensional organization of the genome [53–59]. Chromatin confirmation capture studies provided compelling support for modifications in inter- and intra-chromosomal interactions in breast cancer-compromised cells that are associated with a loss of competency for mammary epithelial cell gene expression that is reciprocally associated with breast cancer initiation and progression. The mammary epithelial cell phenotype is accompanied by transformed and tumor cell-mediated structural and functional properties including the acquisition of properties associated with the hallmarks of cancer, including emergence of cancer stem cells that are competent to autologously develop metastatic tumors.

## 3. The histone code as an epigenetic transcriptional determinant

**Figure 1 •:**
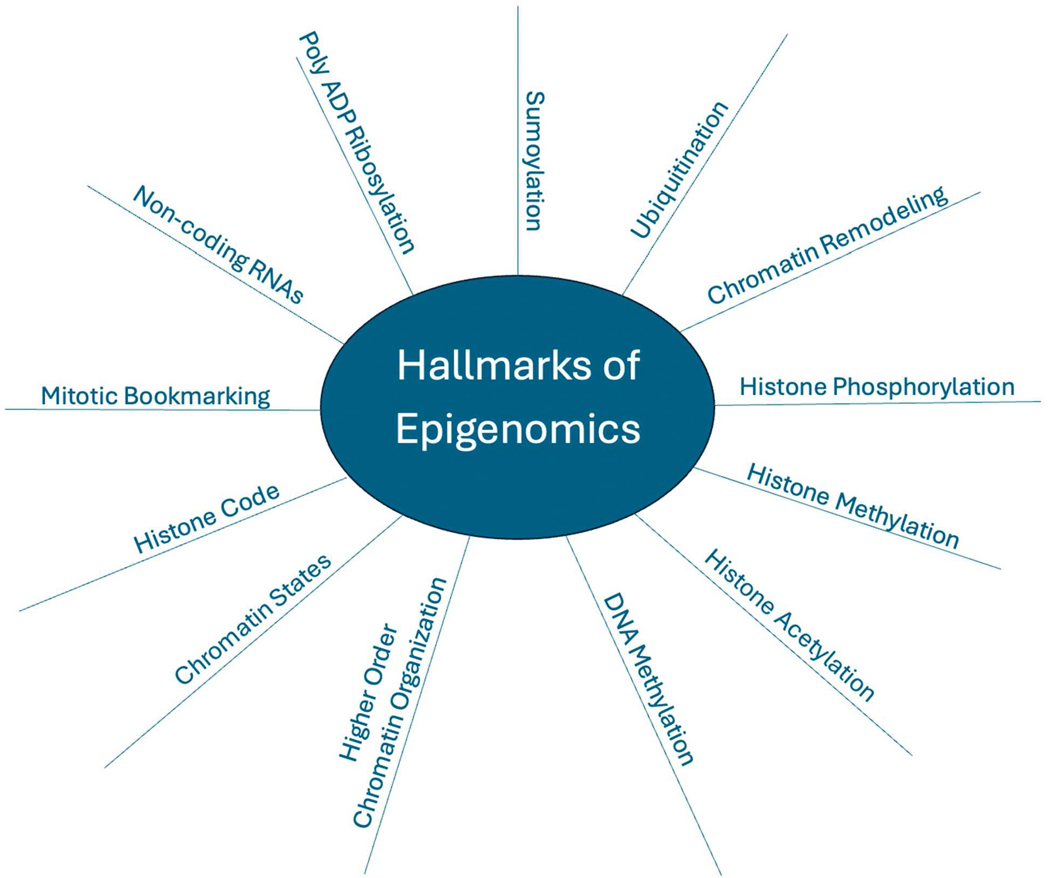
Regulatory parameters of epigenetic control that synergistically function as hallmarks of the epigenome

Evidence is accruing for the combined mechanistic contributions by multiple parameters of genome organization, structure and function to the epigenetic control of gene expression, as summarized in Figure 1. While the regulatory information encoded in DNA remains constant, DNA methylation plays a decisive role in rendering regulatory and structural elements of the genome accessible or refractory to proteins that influence competency for transcription and suppression [60–62]. A significant advance in navigating the complexities of epigenetic control initially emerged two decades ago with the proposal, reinforced by correlations and functional evidence, that the histone-code post-translational modifications of histone proteins mediate protein–DNA and protein–protein interactions to support or repress transcriptional activity [1–12] (Table 1).

From a regulatory perspective, the architectural and functional logistics of the histone code reflect the composite representation and localization of carboxy-terminal histone modifications in specific genome domains. The regulatory impact of the histone code is operative within gene regulatory elements as well as upstream and downstream of mRNA coding regions, providing critical cues for biological control and gene expression in disease-compromised transcriptional regulation. Expanding a mechanistically characterized histone modification includes histone phosphorylation that primarily contributes to transcriptional competency and sustained transcriptional activity. Histone acetylation mechanistically supports transcription. And an extensive series of histone methylation events are emerging as specific mediators of gene activation and suppression (Table 1).

The molecular basis for the participation of modified histones in the epigenetic control of gene expression and regulatory plasticity is broadening the appreciation for genome structure and relationships that are operative for transient and long-term commitments to gene activation or repression as well as for those that epigenetically control gene expression for regulatory windows during development or tissue remodeling. Here, the histone code provides essential regulatory cues for epigenetic control in pluripotent embryonic stem cells and in lineage-committed stem cells that support tissue renewal and regeneration throughout the lifespan continuum.

The scope of regulatory processes that are dependent on epigenetic control by histone modifications include histone–DNA and histone–histone interactions; gene accessibility to transcriptional and coregulatory proteins; noncoding RNA-mediated control; access as well as activities of chromatin remodeling proteins; and higher-order chromatin organization that includes inter- and intra-chromosomal interactions (Table 1).

Compelling insight into the mechanistic basis for the epigenetic control of gene expression by post-translational histone modifications is the scope of modified histones (greater than 40) that contribute to the histone code. Equally relevant is the extensive enzymology supporting physiologically responsive chromatin states, as well as altered chromatin states that are associated with the initiation and progression of acute and chronic disease [2, 63–71]. Beyond enhancing insight into epigenetic mechanisms that are pivotal for mapping epigenetic regulatory networks, the biochemistry of chromatin states that are reflected by the histone code is providing novel therapeutic targets for cancer with increased specificity and reduced off-target effects.

## 4. The epigenetically mediated transcriptional control of the cell cycle and the requirement for the dynamic remodeling of regulatory machinery at a novel checkpoint that is perturbed in cancer

When the study of eukaryotic gene expression was in its infancy, Gary Stein in collaboration with Janet Stein and Andre Van Wijnen had the vision to develop an epigenetically mediated architectural perspective for the control of gene expression as a paradigm for defining transcriptional regulatory mechanisms at the onset of the S phase. Their contributions have been breakthroughs in cell cycle and growth control that are relevant to fundamental regulatory mechanisms for proliferation and directly relate to aberrant gene expression in cancer. Combining in vitro and in vivo experimental approaches, they identified determinants of transcriptional competency at the G1/S phase transition in response to factors regulating cell cycle progression [23, 24, 72–79]. They provided the initial demonstration that transcription factor phosphorylation modulates the cell cycle-dependent control of gene expression [73]. The laboratory cloned the first human cell cycle-regulated genes that serve as blueprints for responsiveness to proliferation-dependent signaling pathways required for S-phase initiation [25]. They were the first to characterize mechanisms, including chromatin remodeling and chromatin–transcription factor interactions, that couple histone gene expression with DNA replication [80–82]. The group advanced the field of cell cycle control significantly when they linked the control of histone gene expression with the organization and assembly of the transcriptional regulatory machinery in nuclear domain-designated histone locus bodies. The group identified novel cell cycle-regulatory proteins [75–79, 83–86] and a checkpoint at the onset of the S phase that is E2F-independent and temporally as well as biochemically and functionally distinct from the restriction point in G1, and pioneered the characterization of restriction point-S phase signaling [83, 84]. They identified a critical regulatory cascade integrating the restriction point with the G1/S phase transition [85, 86]. They characterized histone locus bodies as defined nuclear microenvironments that support competency for DNA replication and physiologically responsive histone gene activation and biosynthesis for packaging nascent DNA into chromatin. Utilizing chromosome confirmation capture approaches, the laboratory discovered modifications in inter- and intra-chromosomal interactions that represent compromised higher-order genomic organization during tumor initiation and progression [35].

While epigenetically mediated transcriptional regulation is obligatory for histone gene expression during the S-phase of the cell cycle, the transcriptional control of histone genes must be coordinately controlled and functionally integrated with the post-transcriptional control of histone messenger RNA processing [71, 87–96] and the regulation of histone messenger RNA stability. The integration of these regulatory parameters is required to couple histone gene expression with DNA replication, support histone biosynthesis during the S phase of the cell cycle and provide histone proteins to package two-and-a-half yards of DNA in every cell into chromatin during the S phase.

## 5. Unique epigenetic mechanisms in the abbreviated pluripotent stem cell cycle

Pluripotent stem cells support the developmental requirement to epigenetically establish three germ layers with a capacity for indefinite proliferation and a competency for differentiation into endoderm, mesoderm and ectoderm. In both humans and mice, pluripotent stem cells have been isolated from blastocysts during the initial stages of development. These cells have provided valuable resources for the identification and characterization of epigenetic regulatory mechanisms that control lineage commitment and the epigenetic regulatory cascades which control differentiation and cell type-specific gene expression. A fundamental question is the regulatory requirements for the control of proliferation and cell growth to support the expansion of pluripotent stem cells under conditions that conserve competency to establish lineage phenotypes that are instrumental for development.

The Stein laboratory established that human embryonic stem cells rapidly self-renew via an abbreviated cell cycle, characterized by a drastically shortened G1 phase [97–101]. In these pluripotent cells, the transcriptional regulatory machinery, including histone locus bodies, is architecturally organized and assembled rapidly following the conclusion of mitosis to support DNA replication that is coupled with the biosynthesis of histone proteins. This discovery, confirmed by studies with induced pluripotent stem cells [97, 102], provides a mechanistic basis for the rapid self-renewal capabilities of pluripotent embryonic stem cells and presents a biological context to relate the nuclear structure and functional control of cell cycle progression. The research team is positioned to mechanistically distinguish pluripotent stem cells (blastocyst-derived and induced pluripotent cells) with unrestricted proliferation and an abbreviated but stringently regulated cell cycle from transformed and tumor cells with unrestricted proliferation by aberrant cell cycle and growth control.

The pluripotent cell cycle is not restricted to human embryonic stem cells. An abbreviated cell cycle with a reduced G1 phase has been observed in mouse embryonic stem cells [103, 104]. It appears that the epigenetic mechanisms that control cell cycle progression during a restricted time period are similar in humans and mice. The overarching mechanism that supports an abbreviated cell cycle in pluripotent stem cells appears to be the constitutive expression of cell cycle-regulatory genes that are expressed in a stage-specific manner following lineage commitment when an expanded G1 period is initiated.

## 6. Cancer-compromised epigenetic control

Epigenetic control contributes to cancer-compromised gene expression as a consequence of two functionally interrelated perturbations in transcriptional regulation. First is the epigenetic downregulation of the regulatory machinery that directly supports the expression of tissue-specific, phenotype-relevant genes and tumor suppressor activity. Second and equally relevant to perturbations in cell structure and function that are associated with the development and progression of a transformed tumor phenotype is the initiation of gene expression which is linked to the expanding cohort of cell properties which are associated with the hallmarks of cancer. Here, epigenetic regulation is pivotal for aberrations in gene expression that include impact on cell cycle and growth control, cell mobility and motility, competency for the recognition of DNA damage and capacity for DNA repair, cell adhesion, the fidelity of metabolic activities, the control of cell survival and activities of oncogenes and tumor suppressors.

The Stein laboratory provided novel insight into epigenetic control in breast cancer, prostate cancer and leukemia biology and pathology. They established the mechanistic requirements of RUNX transcription factor biosynthesis and the fidelity of intra-nuclear trafficking for tissue-specific chromatin states and chromatin remodeling. Utilizing molecular and cellular approaches, the research group identified modified RUNX interactions with target gene-regulatory elements that result in epigenetically altered chromatin structure [13, 14, 105, 106]. Cancer-compromised gene expression, as an epigenetically modified consequence, has been reinforced by functional knockdown studies [107, 108]. Requirements for physiologically responsive inter- and intra-chromosomal interactions by chromosome confirmation capture experiments provide support for the active engagement of higher-order chromatin organization to sustain mammary cell phenotypic gene expression [109–112]. And functional linkage has been demonstrated for higher-order chromatin structures with modified gene expression in early-stage breast cancer cells. These findings provide an additional mechanistic and clinically informative dimension of epigenetic regulation. The research group has developed breast and prostate tumor-derived and circulating epigenetic signatures as potential biomarkers [48, 113].

## 7. The mitotic bookmarking of genes provides a novel epigenetic mechanism for control of cell fate and lineage commitment

The mitotic bookmarking of genes is a novel epigenetic mechanism for transcriptional memory that is obligatory to sustain tissue-specific gene expression through cell division. By this mechanism, regulatory machinery that is organized as focal microenvironments in the interphase nucleus can be retained on target gene promoters through mitosis [17–21]. Phenotypic transcription factors, histone modifications and DNA methylation collectively contribute to bookmarking; they comprise epigenetic signatures that poise genes for activation or suppression following mitosis. These studies provide transformative insight into the dynamic remodeling of nuclear bodies, potentially liquid phase-separated domains, at specific genomic sites through the cell cycle with mechanistic support for regulatory plasticity and modifications with tumorigenesis [17–21]. A novel dimension to the molecular etiology of cancer is mitotic-specific bivalency. Here, histone modifications that functionally characterize pluripotent embryonic stem cells are recapitulated in early-stage tumor cells [49, 50, 114, 115]. Designated as oncofetal epigenetic control, this mechanism has the potential to advance the understanding of the epigenetically driven loss of phenotypic cell function that accompanies tumorigenesis in prostate and breast cancer [49, 50, 114–116].

## 8. Transcription factor targeting to nuclear domains for tissue-specific gene expression

A new dimension to understanding transcriptional control was provided by identifying the first subnuclear trafficking signal that is relevant both in vitro and in vivo for biological control as well as tumor onset and progression [13, 14, 117–119]. This unique targeting sequence, shared by the Runx 1, 2 and 3 transcription factors which can function autonomously and independently of a nuclear input signal, directs AML/CBFβ (Acute Myeloid Leukemia–Core Binding Factor β) regulatory proteins to nuclear matrix-associated subnuclear domains that support transcription. It has additionally been shown that the t [20, 37] chromosomal translocation in AML leukemia that disrupts the AML locus results in the aberrant intra-nuclear trafficking of AML transcription factors and compromised hematopoietic-specific gene expression [120, 121]. These studies established that the fidelity of transcription-factor localization is required for microRNA-mediated activation and the suppression of regulatory networks that are operative in breast and prostate cancer as well as in leukemogenesis [120, 121]. The intra-nuclear trafficking of transcription factors has been functionally linked with the control of tissue-specific gene expression using in vivo genetic approaches [119, 122]. These results are key contributions to understanding the role of nuclear architecture in mechanisms that facilitate the dynamic assembly and activity of transcription complexes for the spatial and temporal control of biological and cancer-compromised gene expression.

## 9. Epigenetically mediated skeletal biology and pathology

Partnering with Jane Lian, Andre van Wijnen and Jonathan Gordon, our research group has prioritized the pursuit of epigenetic regulatory mechanisms that are operative during skeletal development and bone remodeling as well as compromised in skeletal pathology. The research team pioneered the development of cell culture models that were instrumental for mechanistically defining the developmental sequence of gene expression that is required for osteoblast lineage, commitment and differentiation [123–125]. Phenotypic determinants for bone cell proliferation and differentiation were identified and characterized [13, 123–134], with validations in genetically modified in vivo mouse models [135–141]. Leveraging the power of rapidly evolving molecular approaches, the research group provided valuable insight into chromatin remodeling and chromatin states [123, 124, 129] that render skeletal gene-regulatory elements competent for responsiveness to physiological regulatory signals [13, 128, 133, 142–146]. Recognizing the significance of noncoding RNAs as mediators of epigenetic skeletal control, the laboratory has advanced the understanding of the contributions of micro RNAs as well as long noncoding RNAs to epigenetic parameters of skeletal development, tissue renewal, fracture repair and bone metabolic diseases [37, 147–152]. With Rami Aqueilan, progress is being made to understand regulatory defects associated with osteosarcoma [153, 154].

## 10. Conclusion: a perspective for epigenetically mediated biology and pathology

The arbitrary boundaries that defined transcriptional control and epigenetic regulation have evolved to an integrated regulatory paradigm. The emerging perspective is an overarching model for contributions to biological control by the selective utilization of DNA-encoded regulatory information that is complemented by non-DNA-encoded regulatory mechanisms which reside in DNA methylation, chromatin states, higher-order genome organization and regulatory cues provided by noncoding RNAs. Optimism is justifiable for clinical applications with an accelerated mechanistic understanding of cancer-compromised epigenetic control and an expanded availability of epigenetic targeting reagents with high specificity and minimal off-target effects.

## Figures and Tables

**Table 1 •:** Partial list of posttranslational histone modifications that contribute to the histone code, influencing chromatin organization at multiple levels including higher order chromatin structure, and chromatin states that mediate regulatory processes including competency for transcription, poising genes for transcriptional occupation, DNA replication, DNA repair and transcriptional impression. Histone modifications are predominantly reversible by histone deacetylases, histone demethylases, and histone phosphatases, enzymatically supporting plasticity and physiological responsiveness for epigenetic control of gene expression. Green histone modifications indicate engagement in gene activation and red histone modifications indicate engagement in gene repression.

The Biochemistry of the Histone Code
H3K4me1
H3K9me1
H3K27me1
H3K79me1
H4K20me1
H2BK5me1
H3K9me2
H3K27me2
H3K79me2
H3K4me3
H3K9me3
H3K27me3
H3K79me3
H3K79me3
H2BK5me3
H3K9AC
H3K14AC
H3K27AC
H3K122AC
H2BK5AC
H3K36me1
H3K36me2
H3K36me3
H3K23AC
H3K36AC
H3K55AC

## Data Availability

Data supporting these findings are available upon request.
